# The influence of hydrodynamics and ecosystem engineers on eelgrass seed trapping

**DOI:** 10.1371/journal.pone.0222020

**Published:** 2019-09-03

**Authors:** Lukas Meysick, Eduardo Infantes, Christoffer Boström

**Affiliations:** 1 Environmental and Marine Biology, Faculty of Science and Engineering, Åbo Akademi University, Åbo, Finland; 2 University of Gothenburg, Department of Marine Sciences, Fiskebäckskil, Sweden; Helmholtz-Zentrum fur Ozeanforschung Kiel, GERMANY

## Abstract

Propagule dispersal is an integral part of the life cycle of seagrasses; important for colonising unvegetated areas and increasing their spatial distribution. However, to understand recruitment success, seed dispersal and survival in habitats of different complexity remains to be quantified. We tested the single and synergistic effects of three commonly distributed ecosystem engineers—eelgrass (*Zostera marina*), oysters (*Magellana gigas*) and blue mussels (*Mytilus edulis*)—on trapping of *Z*. *marina* seeds in a hydraulic flume under currents. Our results suggest that seed retention increases with habitat complexity and further reveal insights into the underlying mechanisms. In eelgrass canopy, trapping occurred mostly through direct blocking of a seed’s pathway, while trapping in bivalve patches was mainly related to altered hydrodynamics in the lee side, i.e. behind each specimen. With increasing flow velocity (24–30 cm s^-1^ in eelgrass canopy, 18–30 cm s^-1^ in bivalve patches), modifications of the sediment surface through increased turbulence and erosive processes became more important and resulted in high seed trapping rates. Furthermore, we show that while monospecific patches of seagrass and bivalves had different trapping optima depending on flow velocities, intermixing resulted in consistently high trapping rates throughout the investigated hydrodynamic gradient. Our results highlight the importance of positive interactions among ecosystem engineers for seed retention and patch emergence in eelgrass.

## Introduction

Seagrasses can reproduce through a mix of vegetative (rhizome elongation) and sexual (dispersal of propagules) propagation. Clonal growth was commonly suggested to be crucial mostly for proliferation [1] and an *in situ* response to local disturbances [2]. Sexual propagation, on the other hand, plays a substantial role for the demographic and genetic connectivity in seagrass ecosystems [3, 4]. Yet, it has been shown, that this can locally differ considerably. For instance, in Finland, where eelgrass (*Zostera marina* L.) lives at its lower salinity range limits, or along the Portuguese coast where it lives it its upper temperature range limit, clonal proliferation can predominate over sexual reproduction. Here, somatic mutations are the major source of genetic variation [5]. Nonetheless, propagule dispersal is particularly relevant for colonisation of unvegetated areas following potential emergence of new patches [6]. Empirical evidence further suggests that seagrass seed banks can induce large-scale seedling emergence, buffer local disturbances [7] and facilitate recovery and restoration processes [8, 9].

The distance seeds or fruits can travel is species specific and the transport mechanisms include multiple drivers [6, 10]. Rafting of flowering shoots with viable seeds of eelgrass, for example, may cover distances up to 150 km [11, 12] while the negatively buoyant individual seeds are primarily transported as bedload by currents and waves only over a few meters [13]. Besides transport through physical processes, there is also a potential for beneficial biotic transport, indicating that eelgrass seeds can be carried over several kilometres away from their release point source through the guts of vertebrate species [14, 15].

Fragmentation of seagrass habitats [16] and seed predation by fish and crustaceans can considerably reduce seed densities and limit seedling establishment [17, 18]. Particularly, seed predation by the shore crab *Carcinus maenas* has been identified as a potential positive feedback, preventing eelgrass recovery along the Swedish west coast [18]. In contrast, some infaunal species have been shown to promote seed retention and germination rates. Bioturbating polychaetes, for instance, can enhance trapping of eelgrass seeds through modification of the sediment surface [19] and might increase seed burial, thus lowering predation risks [20, 21, 22]. Yet, we still lack a detailed, quantitative understanding of the interaction between seed dispersal and different ecosystem engineers to determine which traits are important for predicting the physical movement of sexual propagules [10].

Bivalves commonly co-occur in seagrass-sand habitats [23, 24]. Despite potential negative interactions (e.g. accumulation of toxic levels of sediment sulphide by bivalves, [25]; and reduced food availability in seagrass meadows, [26]), many studies suggest the co-occurrence of seagrass and bivalves to be mutually beneficial [27, 28, 29]. Seagrasses for example can provide shelter from predators or physical disturbance, while bivalves might increase light penetration through particle filtering. Both are often considered ecosystem engineers [30], altering shallow water hydrodynamics [31, 32, 33, 34] and sediment properties [35, 36]. Hydrodynamic processes and bottom complexity are potentially crucial factors determining dispersal and recruitment of seeds [19, 37, 38], indicating that benthic ecosystem engineers can play a significant role in retention of seagrass seeds.

By manipulating densities of seagrass shoots and bivalves in a hydraulic flume, we tested how the physical arrangement of benthic structures can benefit seed entrapment and thus eelgrass patch emergence under a range of hydrodynamic conditions. Specifically, we aimed to (1) assess eelgrass seed dispersal and trapping by individual and combined synergistic effects of eelgrass shoots, pacific oysters (*Magellana gigas*, previously known as *Crassostera gigas*) and blue mussels (*Mytilus edulis*) and to (2) quantify which flow velocities affect sediment dynamics and seed retention by those ecosystem engineers.

## Methods

### Collection of study organisms

Eelgrass shoots, oysters and blue mussels were collected at Bökevik in the Gullmar Fjord, Sweden (58°25’ N; 11°45’ E), where these species commonly co-occur (Infantes pers. obs.). Permission to harvest eelgrass shoots and bivalves were obtained from the Swedish Administrative Board of Våstra Götaland. Eelgrass seeds were collected by harvesting reproductive shoots at 1–3 m depth in the Gullmars Fjord, Gåsö in Sweden, in July 2017. Reproductive shoots were stored in 1500 L outdoor tanks at Kristineberg until the seeds were released [39]. The seeds were stored until used in the experiments at salinity of 34 PSU and temperature of 5°C to prevent germination [40]. Shoots and bivalves were stored in tanks with flow-through surface water from the fjord. Seed viability was assessed using the fall velocity method [39, 41] according to which firm and intact seeds that have a fall velocity (*w_s_*) ≥ 5 cm s^-1^ are likely to develop into a seedling. We determined fall velocity of each seed in a vertically placed glass tube (50 cm long and 7.5 cm diameter) filled with seawater. Using a conservative estimation, only seeds with *w_s_* ≥ 6 cm s^-1^ were included for further experiments.

### Flume setup and hydrodynamic properties

**Fig 1:**
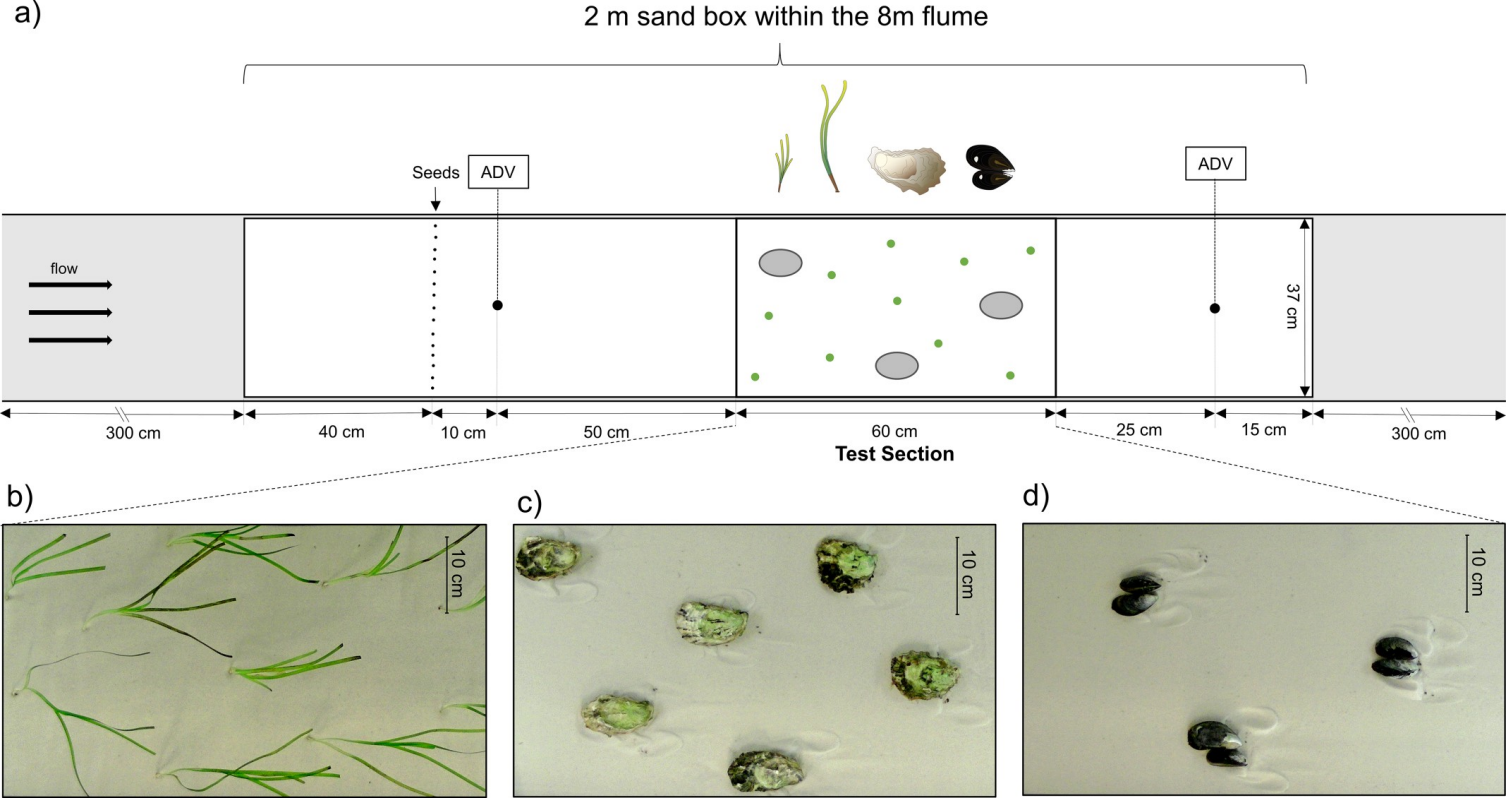
Setup of a) the current flume, and the test section including exemplified b) eelgrass, c) oyster and d) blue mussel treatments from top view perspective. Note that panel a) is not to scale. Symbols from IAN Symbol Libraries.

A unidirectional current flume was used to simulate the dispersal and trapping of seeds at different hydrodynamic conditions. The flume was 8 m long, 0.5 m wide and 0.5 m deep (Fig 1). The flume had a 2 m long and 0.37 m wide test section, which consisted of an embedded box filled with sand. To avoid friction and edge effects, the sandbox is placed 5 cm from the flume wall. For easier detection of seeds and to establish standardized sediment conditions during the experiments, we used artificial Sansibar^®^ WHITE aquarium sand with a 0.2–0.6 mm grain size distribution. The water level was maintained at 15 cm to simulate high flow velocities up to 30 cm s^-1^. This water level does not represent field conditions, but simulates a realistic range of flow velocities at which eelgrass occurs in nature [42]. As this study focuses on bedload transport of eelgrass seeds, we believe that the flow conditions near the sediment surface have a higher relevance, than the overall water level. Unidirectional flow was generated by a motor-run propeller at the far end of the flume controlled by an adjustable speed drive (Dayton Electronic, model 6K119). Flow velocities were measured with an Acoustic Doppler Velocimeter, ADV (Nortek, Vectrino) at a sampling rate of 25 Hz. Horizontal flow velocity used for characterizing the flow conditions in the flume was measured at 5 cm above sediment surface. Vertical profiles of flow velocity were measured at 9 positions (0.3, 1–8 cm above sediment surface at 1 cm intervals) located 50 cm before and 25 cm after the test section for each treatment (Fig 1). Vertical profiles were conducted at 16 cm s^-1^ (hereafter ‘low velocity’) and 30 cm s^-1^ (hereafter ‘high velocity’). Additionally, turbulent kinetic energy (TKE, $0.5\times \left(\bar{{u^{\prime }}^{2}}+\bar{{v^{\prime }}^{2}}+\bar{{w^{\prime }}^{2}}\right)$) was calculated to characterize turbulent conditions within the flume. To assess the effect of each treatment on hydrodynamics near the sediment surface (1 cm), the change in flow velocity (Δ*u* = *u_before_*−*u_after_*) and turbulence (Δ*TKE* = *TKE_before_*−*TKE_after_*) was calculated. A video camera (Kurokesu, C1) was set up at a fixed position 1.5 m above the test section during the whole experiment. After placing a measuring tape in the test section, a photograph was taken capturing the sediment conditions at each trial. Sediment erosion due to turbulent flow behind objects was measured as scouring area by photogrammetry analysis applying the image editing software ImageJ on the photos taken.

### Experimental design

The experiment was conducted during September-October 2017. Within the 2 m test section of the flume, a 60 cm long section was demarcated for placing plants and bivalves (Fig 1). To assess the capacity of biogenic structures to trap eelgrass seeds, 16 treatments were investigated (Table 1). The tested eelgrass shoot densities ranged from 22 to 180 shoots m^-2^. Although these densities represent the lower spectrum of natural meadows in the area [43, 44], they were primarily intended to simulate early stage patch emergence to detect threshold densities potentially facilitating seed entrapment through positive feedbacks. To account for shoot morphology (linked to life history and adaptation to environmental conditions), we used both, narrow and short shoots (~15 cm length, 0.25 cm width), and wide and long shoots (~30 cm, 0.45 cm width). Due to bending of shoots with water movement, the eelgrass canopy was constantly below water surface during the investigated treatments, including those treatments where shoot length exceeded the water level (15 cm).

**Table 1:** Treatments investigated in the flume study. Number of objects corresponds to objects in flume test section. Total width (Tot *w*) corresponds to sum of lateral width of all objects in test section.

Treat-ment	Biogenic structure		No. of obj.	Density (obj m^-2^)	Tot *w* (cm)
1	Eelgrass short	‘low’	5	22.5	1.34
2	Eelgrass short	‘medium’	10	45	2.69
3	Eelgrass short	‘high’	20	90	5.38
4	Eelgrass long	‘low’	5	22.5	2.21
5	Eelgrass long	‘medium’	10	45	4.41
6	Eelgrass long	‘high’	20	90	8.83
7	Eelgrass long	‘very high’	40	180	17.66
8	Oyster	‘low’	1	4.5	5.90
9	Oyster	‘medium’	3	13.5	16.70
10	Oyster	‘high’	6	27	35.00
11	Blue mussel		6	27	19.90
12	Eelgrass short | Eelgrass long		10 | 10	45 | 45	7.10
13	Eelgrass short | Oyster		10 | 3	45 | 13.5	19.39
14	Eelgrass short | Blue mussel		10 | 6	45 | 27	22.59
15	Eelgrass long | Blue mussel		20 | 6	90 | 27	28.73
16	Eelgrass short | Oyster | Blue mussel		10 | 3 | 6	45 | 13.5 | 27	39.29

Oysters and blue mussels co-occur with eelgrass on the Swedish west coast, where they are found in low densities (typically single individuals), rather than dense patches or reefs (Infantes pers. obs.). Thus, investigated treatments for seed trapping ranged between 4.5 to 27 ind. m^-2^, i.e. 1–6 individuals within the test section.

Before every setup, the sediment surface was smoothened to a plane surface to standardize initial sediment conditions. If treatments included eelgrass shoots, those were carefully pushed into the sediment. In sandy habitats on the Swedish west coast, oysters are usually orientated with the planar area towards the bottom and not erect as for muddy sediments (Infantes pers. obs.). Thus, all bivalves were placed on top of the sediment pointing towards flow direction mimicking field conditions. Bivalves were orientated in staggered and fixed, non-randomized arrangements to attain maximum surface areas exposed to the currents and to allow for comparison between treatments. Oysters were placed individually in the test section. Blue mussels were orientated in staggered patches of two individuals to ensure that mussels were relatively stable and did not move even when high flow velocities were applied, but also resembled a natural configuration. Seed entrapment capacity for every treatment was determined under 10 current velocities (12–30 cm s^-1^, in 2 cm s^-1^ intervals). Flow was initialized for 2 min beforehand each trial (treatment × velocity) to establish laminar conditions in the flume. Subsequently, thirty seeds were released with a tweezer 60 cm upstream the test section on top of the sediment along the full width of the flume. The number of seeds trapped within the test section was counted and the trapping location of each seed was recorded. Initial pilot tests to characterize seed movement at different flow velocities showed that for a smoothened sediment surface and without additional structure, all seeds passed the test section, independent of the 10 investigated flow velocities.

### Statistical analysis

Binomial logistic regressions were applied to compare trapping success within plant and oyster treatments. A three-way factorial design with shoot density (low: 23 m^-2^, medium: 45 m^-2^, high: 90 m^-2^) and shoot width (short shoots: 2.69 mm, long shoots: 4.41 mm) as continuous predictors and current velocity as factor was used for the plant model. A two-way factorial design with oyster density (low, medium, high) as continuous predictor and flow velocity as a factor was used for the oyster model. Flow velocity was included as factor with three levels (low: 12, 14 cm s^-1^; medium: 20, 22 cm s^-1^; high: 28, 30 cm s^-1^) instead of a continuous variable, due to non-exponential relation between current velocity and seed trapping. Thereby two velocities were pooled together to increase the number of replicates from 30 to 60 and thus achieve a more robust statistical output. Model selection was based on minimal Akaike information criterion (AIC) and Chi square tests, excluding non-significant model-terms. Regression based methods were used to relate the scouring area to propagule trapping for each current velocity separately and to test for the effect of object width on flow dynamics and sediment scouring. To meet assumptions for regression analysis, Spearman tests for appropriate weighting and Shapiro-Wilk tests for normality of residuals were conducted. In cases assumptions were not met, data was appropriately transformed (log and square root transformation).

## Results

**Fig 2:**
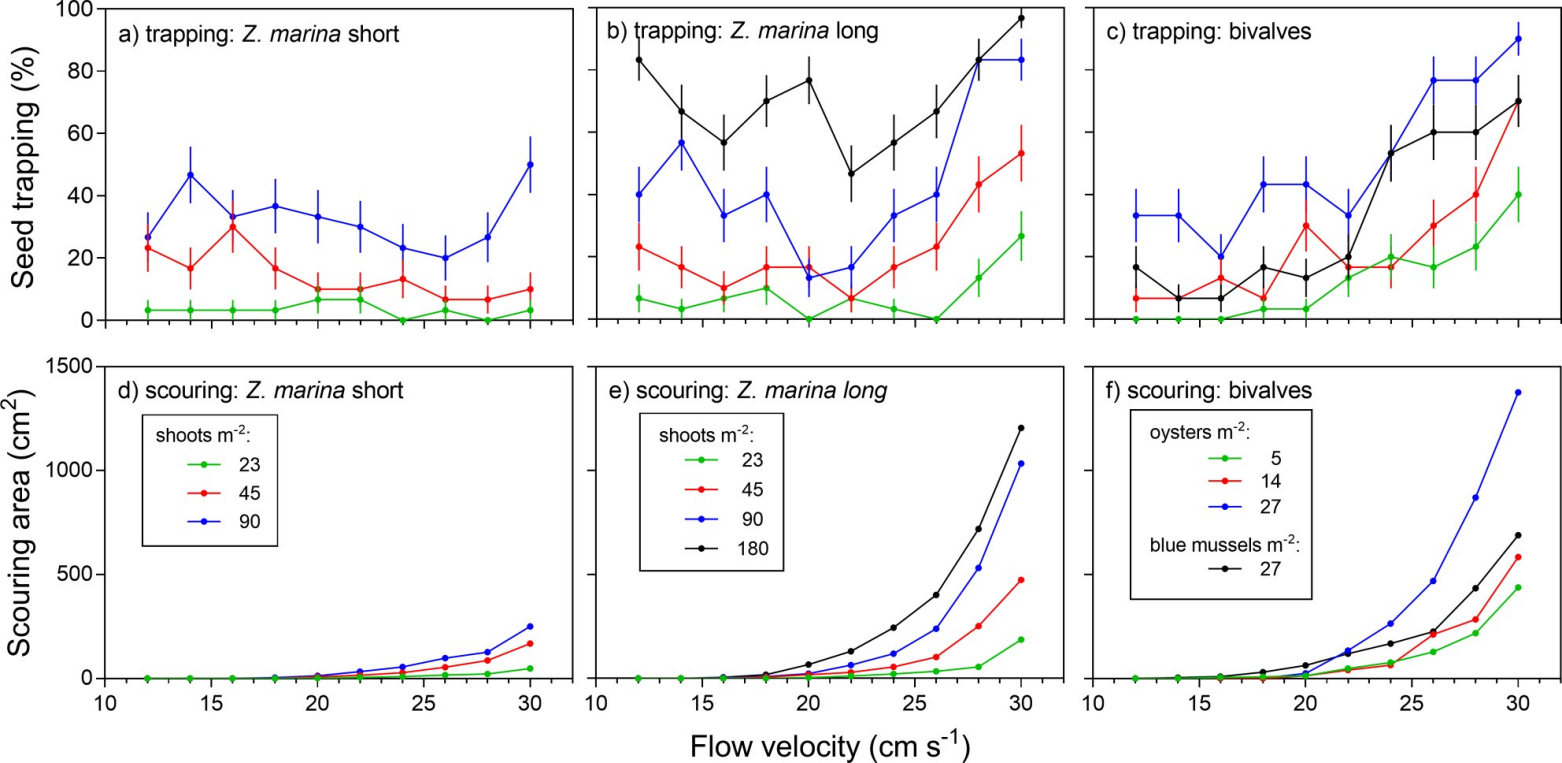
Trapping of eelgrass seeds (Mean ± SE, n = 30) and size of corresponding scouring patterns in different biogenic structures in relation to flow velocity; a), d) short shoots (< 15 cm); b), e) long shoots (> 25cm) and c), f) bivalves.

Short shoots (Fig 2A) were comparably ineffective (<10%) in trapping seeds throughout the tested range of flow velocities at the low shoot density treatment (23 shoots m^-2^). At the medium shoot density treatment (45 shoots m^-2^), seed trapping in short shoots ranged from 5% to 30% with lowest trapping at high flow velocities. At the high shoot density treatment (90 shoots m^-2^), trapping of seeds was between 20% and 50% with highest seed retention at high flow velocity. Long eelgrass shoots (Fig 2B) showed similar success in seed trapping at lower current speeds for respective shoot densities. However, other than for short shoots, an increase in seed trapping towards higher flow velocities could be detected already for low and medium shoot densities. At the highest flow velocity (30 cm s ^-1^) seed retention was 25% at low, 55% at intermediate and 85% at high shoot density, respectively. An additional treatment with 180 shoots m^-2^ showed a further increase in trapping success with 50–95% of seeds trapped (Fig 2B). Logistic regression showed that flow velocity and canopy characteristics (shoot density and shoot width) significantly influenced seed trapping in eelgrass treatments (S1 Table). Additionally, there was an interaction effect of flow velocity and shoot width.

In contrast to eelgrass treatments, all oyster treatments showed an increase in trapping with increasing flow velocity (Fig 2C). Trapping ranged from 0–40% at low oyster density (5 ind. m^-2^), 10–70% at medium density (5 ind. m^-2^) and 20–90% at high density, depending on the flow velocity. The blue mussel treatment (27 ind. m^-2^) showed comparable results to the medium density oyster treatment (Fig 2C). Trapping by oysters was significantly influenced by both oyster density and flow velocity (S2 Table).

Photogrammetry analysis of the sediment surface showed that scouring patterns started to emerge around shoots and bivalves at specific flow velocities (Fig 2D–2F). Once sediment started eroding, the scouring area increased exponentially with flow velocity in each of the treatments (S3 Table). When examined across all treatments and given constant flow velocity (≥ 16 cm s^-1^), regression analysis further showed that scouring area also increased linearly with total lateral width of all specimens (S4 Table, S1 Fig).

**Fig 3:**
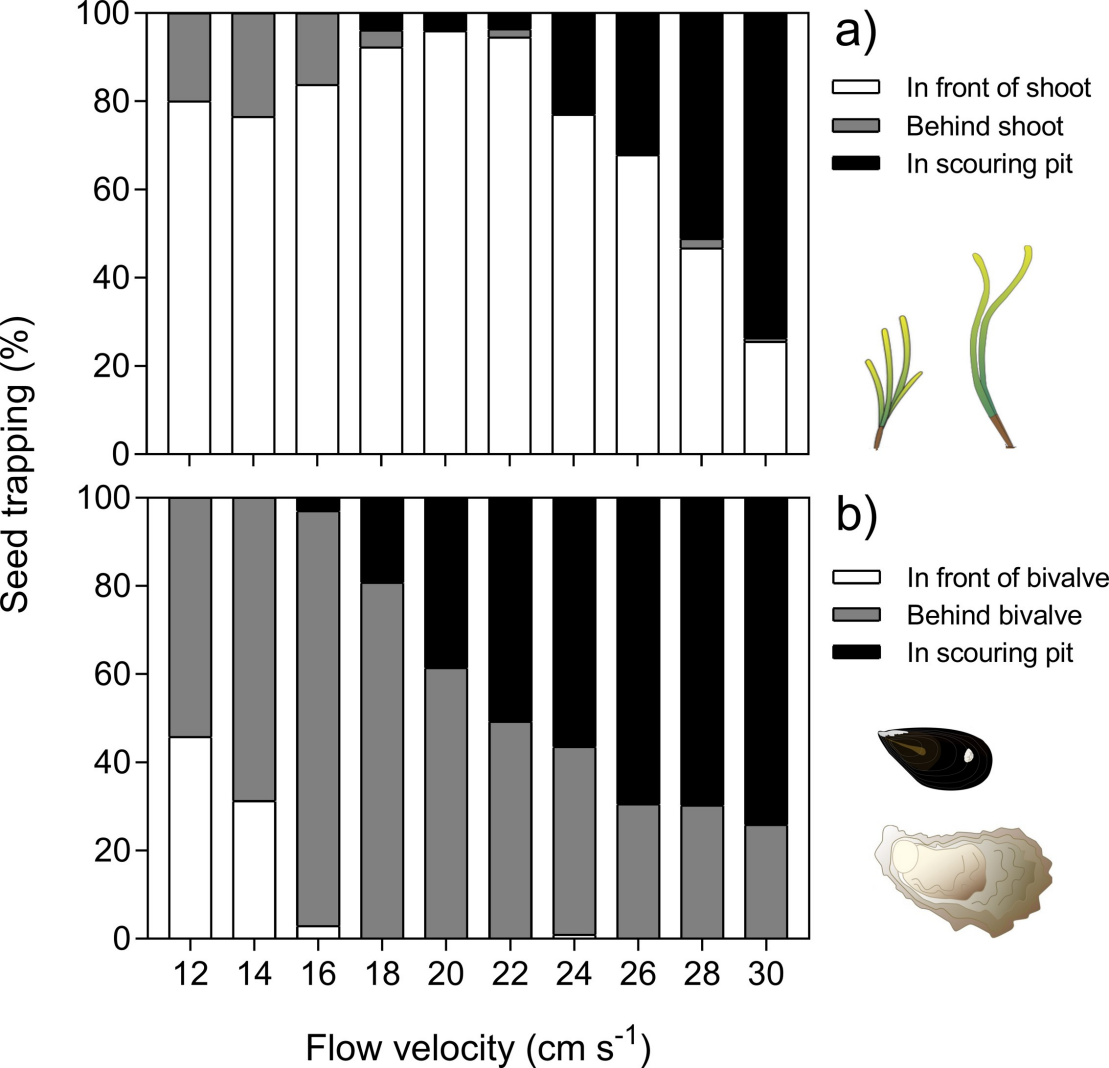
Location of trapped seeds by a) eelgrass and b) bivalves (oysters and blue mussels) at different current velocities (averaged across densities). Symbols from IAN Symbol Libraries.

**Fig 4:**
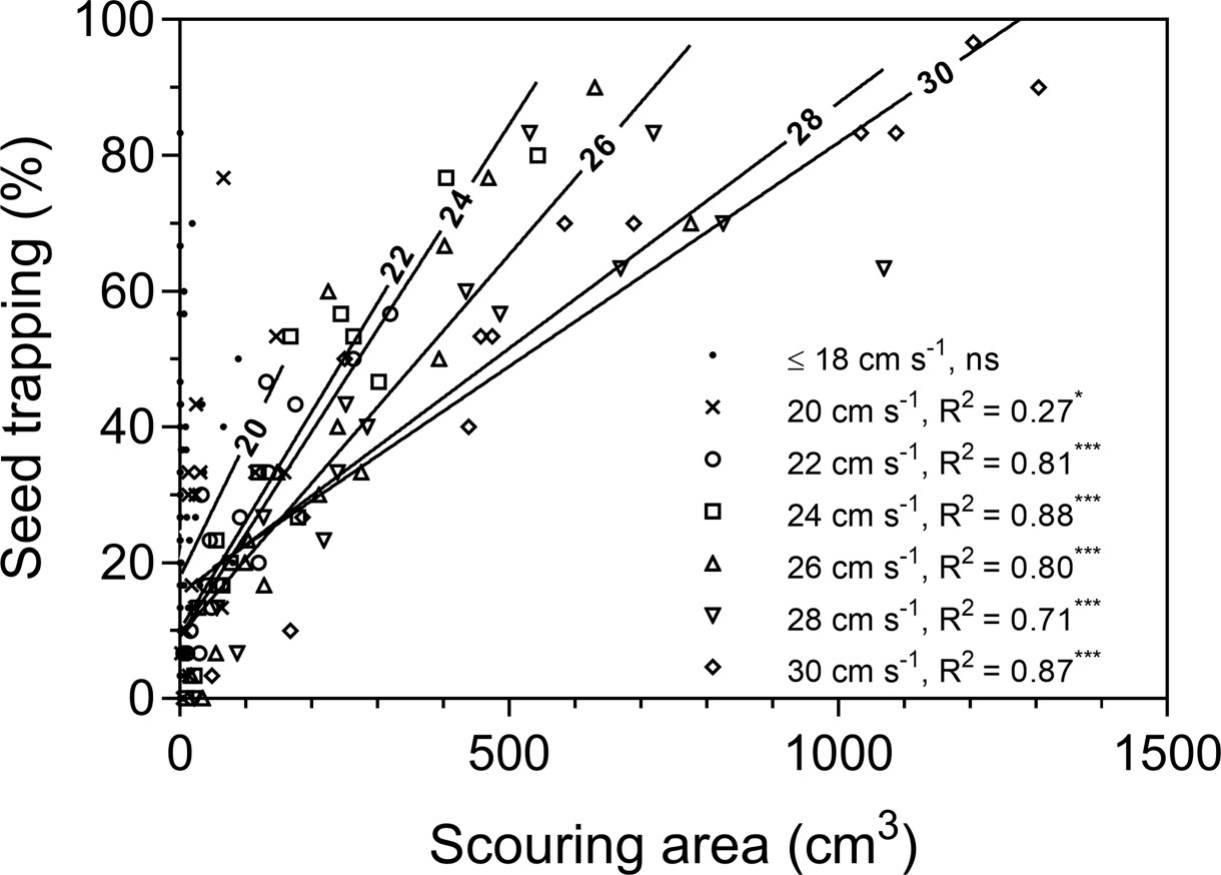
Percentage of eelgrass seeds trapped in relation to the scouring area for different current velocities. Regression lines are indicating that at high current velocities higher scouring areas are needed to result in similar proportions of trapped seeds (decrease in slope). Statistically significant for slope different from zero, is indicated by ^ns^p>0.1, *p<0.05, ***p<0.001.

**Fig 5:**
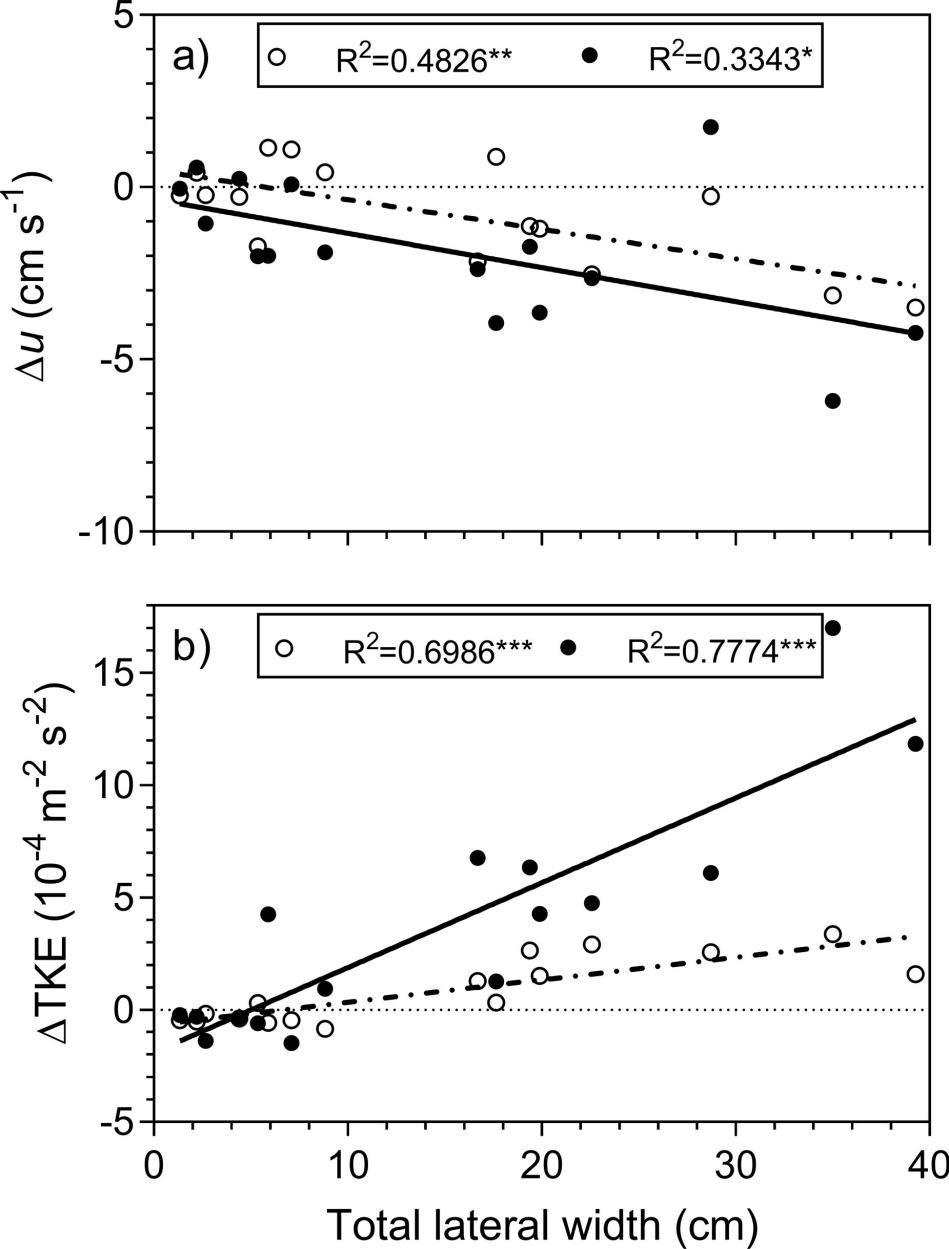
Effects of total width of objects placed within the test section on a) flow velocity, Δu, and b) turbulent kinetic energy, ΔTKE, 1 cm above the sediment. Dashed and solid lines correspond to surface flow velocity of 16 cm s^-1^ and 30 cm s^-1^, respectively. Statistically significant is indicated by *p<0.05, **p<0.01, ***p<0.001.

The position where seeds were trapped changed with underlying hydrodynamics. In eelgrass, trapping was mainly in front of the shoots under lower flow velocities (12–22 cm s^-1^). With increasing flow, trapping in and around scouring pits became more important and accounted for 75% trapping at surface flow of 30 cm s^-1^ (Fig 3A). In the bivalve treatments, the effects of scouring were more pronounced and occurred already at lower flow velocities (Fig 3B). Linear regression further showed that across treatments the scouring area, i.e. sediment complexity, became a significant predictor for seed trapping for flow velocities equal or above 20 cm s^-1^ (Fig 4). The slope of the regression lines thereby decreased with increasing velocity. Below 20 cm s^-1^, seed trapping was not related to sediment scouring, as scouring patters were not large enough for seed retention. Across all treatments, the total lateral width of specimens placed within the test section significantly affected both flow velocity and TKE above the sediment surface (1 cm). The difference in flow velocity, Δ*u*, decreased with total width (Fig 5A). In contrast, the difference in turbulence, Δ*TKE*, increased with total lateral width (Fig 5B). Both effects were more pronounced at a flow velocity of 30 cm s^-1^ than at 16 cm s^-1^. Vertical velocity and turbulence profiles in front and behind the test section of each treatment are summarized in S2 Fig.

**Fig 6:**
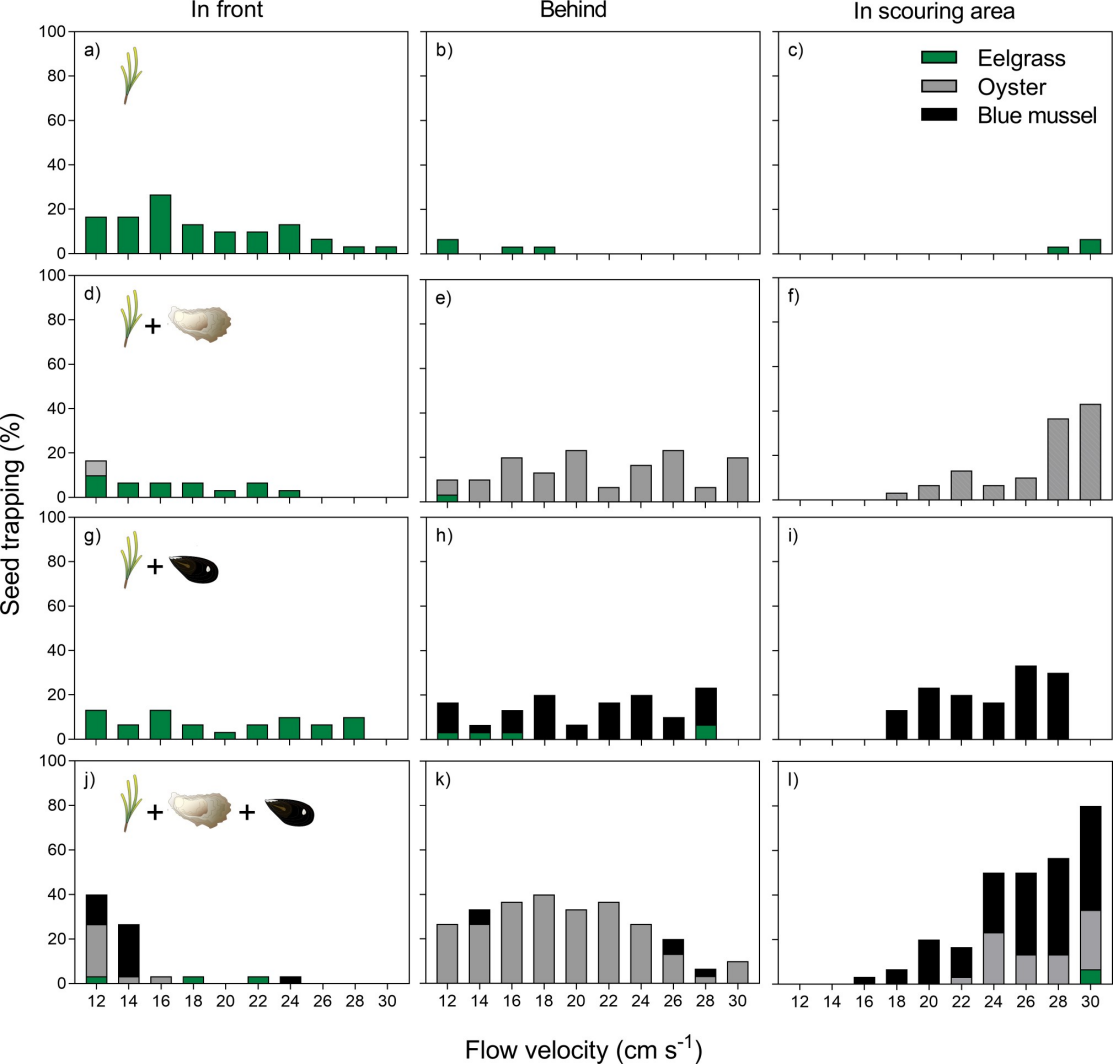
Position of seed entrapment for a)-c) short eelgrass shoots (45 shoots m^-2^), d)-f) intermixing of eelgrass shoots (45 shoots m^-2^) and oysters (14 ind. m^-2^), g)-i) intermixing of eelgrass shoots (45 shoots m^-2^) and blue mussels (27 ind. m^-2^) and j)-l) intermixing of eelgrass shoots (45 shoots m^-2^), oysters (14 ind. m^-2^) and blue mussels (27 ind. m^-2^) for different surface flow velocities (Note: missing information at 30 cm s^-1^ in panel g-i). Symbols from IAN Symbol Libraries.

Intermixing of plants and bivalves had a cumulative effect on propagule trapping, but also affected the trapping positions at different flow velocities. For short shoots (45 shoots m^-2^), trapping was generally low at high flow velocities and scouring played a minor role (Fig 6A–6C). The addition of oysters (14 ind. m^-2^, Fig 6D–6F) or blue mussels (27 ind. m^-2^, Fig 6G–6I) had little effect on trapping success at low flow velocities, but increased trapping towards higher flow velocities. Trapping was caused mostly due to sediment scouring around the bivalves, which accounted for about 70% (oysters) and 50% (blue mussels) seed trapping at maximum flow velocity of 30 cm s^-1^. Seed trapping directly behind bivalves was comparably low (5–25%) without clear trend throughout the flow velocity gradient. Combining all ecosystem engineers (eelgrass, oysters and blue mussels) resulted in high trapping rates at all flow velocities (Fig 6J–6L). In this treatment, eelgrass shoots had a minor effect on trapping, while oysters were mostly responsible for direct trapping under low current velocities and blue mussels for indirect trapping in scouring pits under high current velocities.

## Discussion

**Fig 7:**
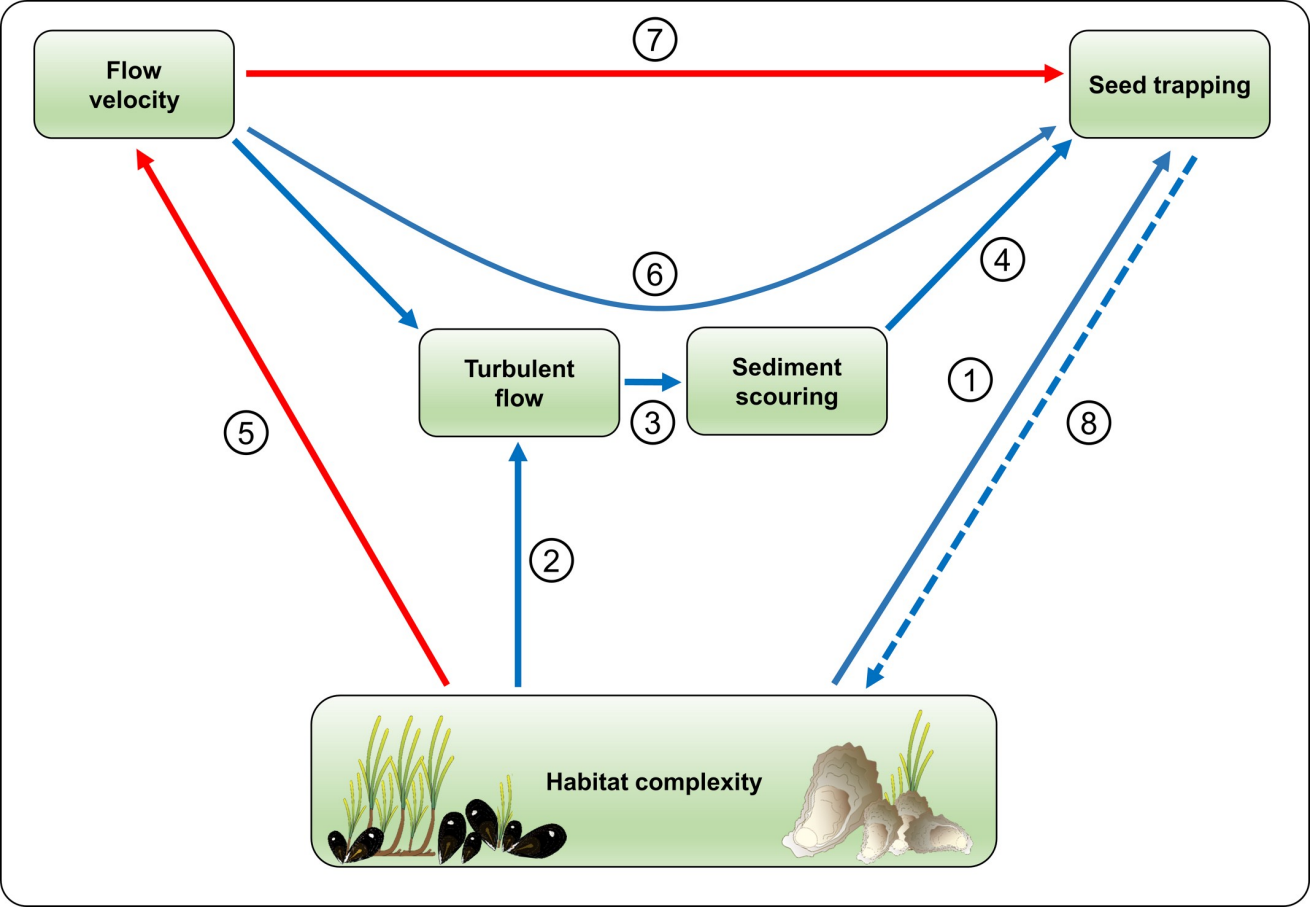
Conceptual diagram on interactions between habitat complexity, sediment scouring, flow velocity and seed trapping. Red arrows indicate negative effect, blue arrows indicate positive effect. Solid arrows correspond to direct effect; dashed arrows correspond to indirect and potential effect. (1) Trapping of eelgrass seeds increases with epibenthic complexity provided by e.g. eelgrass canopy or bivalves (Fig 2; S1 Table; S2 Table); (2) Turbulent conditions increase with complexity of the system (Fig 5B); (3) An increase in turbulence can cause erosion processes [36, 48], increasing surface complexity; (4) Surface complexity increases seed trapping (Fig 4); (5) Epibenthic complexity negatively affects flow velocity (Fig 5A); (6) Flow velocity increases the effect of habitat complexity on turbulent flow (Fig 5B) and sediment scouring (Fig 2D–2F; S4 Table), thus contributing indirectly to seed trapping; (7) The direct effect of flow velocity on seed trapping is assumed negative (Fig 4, [38]); (8) As there is a density dependent effect of eelgrass shoots on seed trapping, while germination success of seeds is independent of density [56], we suggest the existence of a positive feedback over time, facilitating eelgrass patch emergence. Symbols from IAN Symbol Libraries.

We quantified the individual and combined effects of three ecosystem engineers on dispersal and trapping of eelgrass (*Zostera marina*) seeds. Consistent with previous studies, we show that retention of seagrass propagules is dependent on the *in situ* habitat complexity and the prevailing hydrodynamic regime [19, 37, 38]. Monospecific patches of eelgrass and bivalves (*Magellana gigas*, *Mytilus edulis*) showed different trapping rates depending on flow velocities. In sparse eelgrass, our results indicate a negative effect of flow velocity on seed retention. However, denser eelgrass and bivalve treatments increased the turbulence and sediment erosion, which increased trapping success and facilitated seed burial even at high flow velocities. The co-occurrence of these engineers resulted in consistently high trapping rates throughout the investigated hydrodynamic gradient. Our findings provide a detailed understanding of how epibenthic species interact with seed movements and the underlying processes (conceptualized in Fig 7). Below we firstly discuss the three main mechanisms involved in the trapping process and the effects of flow, secondly we outline potentially important feedback mechanisms, and finally discuss the implications of seagrass-bivalve interaction for seed dispersal under different hydrodynamic regimes.

Eelgrass canopies can facilitate seed retention through direct blocking of a seed’s pathway, particularly at low flow velocities. Bivalves on the other hand seemed comparably ineffective in trapping at low flow velocities, as seeds were mostly dragged around them. With increasing flow velocity other processes became more important for seed trapping, i.e. ecosystem engineering.

We showed that turbulence increased with epibenthic complexity and flow velocity [45, 46]. If strong enough, the resulting reverse flow behind the organisms [47] trapped seeds at the sheltered lee side. This trapping mechanism was particularly important in treatments involving bivalves, which caused much larger turbulences compared to eelgrass shoots at similar flow velocity.

An increase in turbulence also initiated erosion processes, which in turn created distinct, three-dimensional sediment scouring patterns around the organisms [36, 48]. So far, sediment erosion has mainly been considered detrimental in early life stages of eelgrass, since it can dislodge or bury seedlings [49, 50, 51]. However, we showed that scouring could actually benefit seed trapping, by increasing the surface complexity of the sediment. In fact, linear regression revealed that at flow velocities ≥20 cm s^-1^, seed trapping was directly related to total scouring area. Sediment topography is a crucial factor for the bedload transport of seagrass seeds, as micro-topographic depressions can accumulate seeds and form seed banks [37]. In particular, small- and large-scale sediment modifications by ecosystem engineers (e.g. polychaetes and dugongs) can contribute to retention of seagrass seeds [19, 37]. To our knowledge, this has not been demonstrated for conspecifics and epifaunal bivalves. The critical flow velocity to initiate erosion processes was lower in treatments with high total lateral width, due to higher turbulences. Thus, bivalves reached scouring areas large enough for seed entrapment already at lower flow velocities. A positive side effect of trapping through scouring was that seeds additionally became buried under the accumulating sediment. Germination success is likely to be enhanced when seeds are covered by a sediment layer of between 1–5 cm [40, 51, 52]. Furthermore, seed burial might decrease predation loss [18] and prevent seeds from being washed away by currents [13]. Ecosystem engineers such as infaunal mussels and polychaetes facilitate seed burial through sediment reworking [20, 22, 53]. The effect on seed development, however, is suggested context dependent, as the positive germination success is reduced when sediment cover exceeds 5 cm [51]. As we did not specifically measure burial depth, predictions for the effect of sediment scouring on eelgrass seed germination are only hypothetical. Yet, for the investigated flow velocities, scouring patterns did not exceed 2–3 cm in height, and thus are unlikely to reduce germination.

Flow reduction caused by different substratum has been shown to be important for propagule trapping [38]. Our results consistently show that near bottom flow velocities decreased with increasing epibenthic complexity. However, as the investigated densities of eelgrass shoots and bivalves were rather low, the resulting flow reduction was generally minor. Separating the effect of flow velocity on seed trapping, by simultaneously disregarding sediment scouring remained difficult in our study, as scouring processes where directly related to the hydrodynamic regime. Seeds were entirely trapped in front, behind or in scouring scars caused by plants and bivalves. Thus, there was not a direct relation between flow velocity and seed trapping. Yet, with increasing flow velocities, a higher sediment complexity was needed to trap a similar proportion of seeds (see Fig 4), suggesting that the sole effect of flow velocity on seed trapping is negative (Fig 7).

Ecological feedbacks are important for patch emergence and resilience in seagrass ecosystems, but can also prevent natural recovery processes [54, 55]. Our results show that the capacity of an eelgrass patch to entrap and retain seeds is positively related to both, shoot density and shoot size, i.e. the denser and the wider the shoots are, the more seeds can potentially be trapped (Fig 2A and 2B, S1 Table). Seed germination success is independent of seed density [56], indicating that a higher number of trapped seeds in a distinct area can increase the number of seedlings and positively contribute to population establishment. This suggests the existence of a self-reinforcing feedback over time, potentially facilitating early establishment and patch emergence in eelgrass ecosystems (Fig 7). Such feedback mechanisms can be enhanced by the beneficial effect of epibenthic structures on hydrodynamics (decreased flow velocity, increased turbulence) and sediment scouring. However, our results also show that at sparse shoot densities, trapping is almost negligible, particularly when shoots are short, implicating context dependent shoot recruitment *in situ*.

The outcome of interactions between plants and bivalves can be highly context dependent and some studies indicate negative effects, such as sulphide accumulation or reduced food availability [25, 26, 57]. However, plant-bivalve associations can also be mutually beneficial [28, 29]. Oysters and blue mussels, for instance, can facilitate seagrasses as allogenic engineers [30], by increasing sediment nutrition through deposition [58] or increasing light penetration through particle filtering [59]. Our results indicate that bivalves can also play a positive role as autogenic engineers by facilitating seed trapping. Thus, bivalves can potentially contribute to overcome density-dependent thresholds to initiate positive feedbacks for eelgrass succession (Fig 7). Particularly at high flow velocities where sparse eelgrass patches are poor in seed retention, our results indicate high entrapment by bivalves.

As the combination of eelgrass, blue mussels and oysters resulted in the highest trapping rates, this study further highlights the importance of intermixed habitats. Monospecific patches of bivalves and eelgrass had different trapping optima depending on flow velocities. Short eelgrass shoots where comparably inefficient at high flow velocities, as scouring areas remained small due to small shoot width [36]. Bivalves, on the other hand, showed the lowest trapping success at low flow velocities, as reverse flow was low and seeds were carried past the bivalves. However, intermixing of both habitat types resulted in consistently high trapping rates throughout the investigated hydrodynamic gradient (>20% trapping, for all flow velocities, except for 14 cm s^-1^).

Experimental results are often difficult to extrapolate to the ecosystem scale [60, 61]. However, given the limited research on seed dispersal and trapping to date, robust and focussed experimental evidence remains an important first step. This flume experiment differed in some respects from natural conditions. For instance, the water level was kept at 15 cm to generate high flow velocities. Furthermore, due to the use of artificial sediment and the absence of microphytobenthos, sediment erosion thresholds might be lower than found for sediment of similar grain sizes in the field [62]. Thus, this study might overstate the effect of the tested biogenic structures on the sediment topography at given flow velocities and time of exposure. However, this study overcame several confounding factors influencing flow in the field, thus ensuring accurate flow measurements and tracking of seeds under control conditions. The design further enabled collection of a large number of replicates to single out the effects of hydrodynamics and habitat complexity on dispersal and trapping of eelgrass seeds, which otherwise remain challenging to gather from measurements in the field.

Besides rafting of flowering shoots [11, 12] or biotic transport [14, 15], bedload transport of seeds is just one way of seed dispersal and important only in the vicinity of seed release [13]. Thus, *in situ* biogenic complexity and sediment micro-topography do not necessarily reflect the potential dispersal distance from a seagrass meadow. Rather they determine the fate of a seed that either passes this area via bedload transport from nearby or arrives from distant meadows after being dropped from a seedpod or a biotic carrier, through seed retention and burial. Moreover, reproductive strategies of eelgrass do locally differ and in areas like the Northern part of the Baltic Sea, were eelgrass lives at its distribution limit due to low salinities, sexual propagation through seeds is almost negligible [5]. Therefore, we conjecture that the strength of the facilitative effect of bivalves, and the associated positive feedback for patch emergence increase in relevance in areas where seed dispersal dominates over clonal proliferation.

Seagrasses, including eelgrass meadows, are facing worldwide declines throughout their distributional range, calling for profound restoration strategies [63], more specifically for eelgrass: [64, 65]. Seeds are frequently used in eelgrass restoration, but seedling establishment is often low due to seed predation and transport through currents [18, 41]. We have shown that eelgrass and bivalves can facilitate retention and burial of seeds even under strong currents. This suggest that site selection for restoration efforts with seeds should carefully consider both co-occurring engineering species and the hydrodynamic regime.

## Supporting information

S1 Table Results of a three-way generalized linear model (binomial distribution with logarithmic link function) describing seed trapping in *Z. marina* patches, with shoot length and shoot density as continuous variables and flow velocity as factor (velocities were pooled as follows: “low” = 12 and 14 cm/s, “medium” = 20 and 22 cm/s, “high” = 28 and 30 cm/s).
Statistically significant is indicated by *p<0.05, ***p<0.001.
(PDF)

S2 Table Results of a two-way generalized linear model (binomial distribution with logarithmic link function) describing seed trapping in oyster patches, with oyster density (5, 14, 27 ind m^-2^) as continuous variable and flow velocity as factor (velocities were pooled as follows: “low” = 12 and 14 cm/s, “medium” = 20 and 22 cm/s, “high” = 28 and 30 cm/s).
Statistically significant is indicated by ***p<0.001.
(PDF)

S3 Table Results of linear regressions of total lateral object width and sediment scouring at different surface flow velocities. (PDF)

S4 Table Results of exponential regressions of flow velocity and sediment scouring at different total lateral object width. (PDF)

S1 Fig Linear regression of scouring area in relation to total lateral width of all specimens in each treatment at 26 cm s^-1^, (‘p < 0.001, r^2^ = 0.878’). (TIF)

S2 Fig Vertical profiles of flow velocity (U) after test section for different densities of short shoots, large shoots and bivalves at surface flow velocity of 16 cm s^-1^ (a-c) and 30 cm s^-1^ (d-f); and vertical profiles of turbulent kinetic energy (TKE) at 16 cm s^-1^ (g-i) and 30 cm s^-1^ (j-l). Dashed lines indicate control vertical profiles in bare sand. Symbols courtesy of the Integration and Application Network, University of Maryland Center for Environmental Science (http://ian.umces.edu/symbols/).
(TIF)

S1 Data Raw data on seed trapping and trapping locations for different treatments. (XLSX)

S2 Data Raw data on velocity profiles for different treatments. (XLSX)
