# Supplementary material for: The influence of hydrodynamics and ecosystem engineers on eelgrass seed trapping
Source: PLoS One. 2019 Sep 3;14(9):e0222020. doi: 10.1371/journal.pone.0222020 (PMC6719863; doi:10.1371/journal.pone.0222020)
Supplement: S1 Table — Results of a three-way generalized linear model (binomial distribution with logarithmic link function) describing seed trapping in Z. marina patches, with shoot length and shoot density as continuous variables and flow velocity as factor (velocities were pooled as follows: “low” = 12 and 14 cm/s, “medium” = 20 and 22 cm/s, “high” = 28 and 30 cm/s). Statistically significant is indicated by *p<0.05, ***p<0.001. (PDF) [file pone.0222020.s001.pdf]

| Term | Df | Dev. | Resid. Df | Resid. Dev | Pr(>Chi) |
|---|---|---|---|---|---|
| Null model comparison | | | 1259 | 1547.8 | <0.001*** |
| Velocity | 2 | 46.2 | 1257 | 1501.6 | <0.001*** |
| Shoot width | 1 | 81.6 | 1256 | 1420 | <0.001*** |
| Shoot density | 1 | 253.8 | 1255 | 1166.2 | <0.001*** |
| Velocity : Shoot width | 2 | 43.0 | 1253 | 1123.2 | <0.001*** |
