# Supplementary material for: The influence of hydrodynamics and ecosystem engineers on eelgrass seed trapping
Source: PLoS One. 2019 Sep 3;14(9):e0222020. doi: 10.1371/journal.pone.0222020 (PMC6719863; doi:10.1371/journal.pone.0222020)
Supplement: S2 Table — Results of a two-way generalized linear model (binomial distribution with logarithmic link function) describing seed trapping in oyster patches, with oyster density (5, 14, 27 ind m-2) as continuous variable and flow velocity as factor (velocities were pooled as follows: “low” = 12 and 14 cm/s, “medium” = 20 and 22 cm/s, “high” = 28 and 30 cm/s). Statistically significant is indicated by ***p<0.001. (PDF) [file pone.0222020.s002.pdf]

Term	Df	Dev	Resid. Df	Resid. Dev	Pr(>Chi)
Null model comparison			539	669.6	<0.001***
Velocity	2	86.3	537	583.3	<0.001***
Oyster density	1	78.0	536	505.3	<0.001***
Velocity:Oyster density	1	5.7	534	499.6	0.058
