# Supplementary material for: The influence of hydrodynamics and ecosystem engineers on eelgrass seed trapping
Source: PLoS One. 2019 Sep 3;14(9):e0222020. doi: 10.1371/journal.pone.0222020 (PMC6719863; doi:10.1371/journal.pone.0222020)
Supplement: S3 Table — (PDF) [file pone.0222020.s003.pdf]

u (cm s^{-1})	slope	intercept	R^2	P
12	0.002	0.005	0.030	0.5244
14	0.138	-0.453	0.229	0.0609
16	0.704	-2.346	0.487	0.0026**
18	1.743	-4.641	0.495	0.0023**
20	3.177	-1.811	0.563	0.0008***
22	6.825	-5.562	0.799	<0.0001***
24	11.59	-8.255	0.834	<0.0001***
26	17.06	10.02	0.878	<0.0001***
28	25.87	53.13	0.859	<0.0001***
30	31.91	232.1	0.685	0.0005***
