# Supplementary material for: The influence of hydrodynamics and ecosystem engineers on eelgrass seed trapping
Source: PLoS One. 2019 Sep 3;14(9):e0222020. doi: 10.1371/journal.pone.0222020 (PMC6719863; doi:10.1371/journal.pone.0222020)
Supplement: S4 Table — (PDF) [file pone.0222020.s004.pdf]

Tot *w* (cm)	slope	intercept	R^2
1.34	0.2964	0.0194	0.986
2.69	0.3021	0.08373	0.998
5.38	0.2662	8.83E-05	0.989
2.21	0.4849	0.01287	0.984
4.41	0.3508	0.0255	0.998
8.83	0.3539	0.3179	0.999
17.66	0.275	0.03969	0.999
5.90	0.31	0.04007	0.997
16.70	0.3195	0.05113	0.989
35.00	0.3518	0.4216	0.984
19.90	0.2467	0.06146	0.995
7.10	0.2969	0.1705	0.999
19.39	0.2844	0.9645	0.995
22.59	0.2341	2.691	0.997
28.73	0.2062	6.282	0.984
39.29	0.1802	0.0194	0.971
