# Supplementary figures and images for: The influence of hydrodynamics and ecosystem engineers on eelgrass seed trapping
Source: PLoS One. 2019 Sep 3;14(9):e0222020. doi: 10.1371/journal.pone.0222020 (PMC6719863; doi:10.1371/journal.pone.0222020)

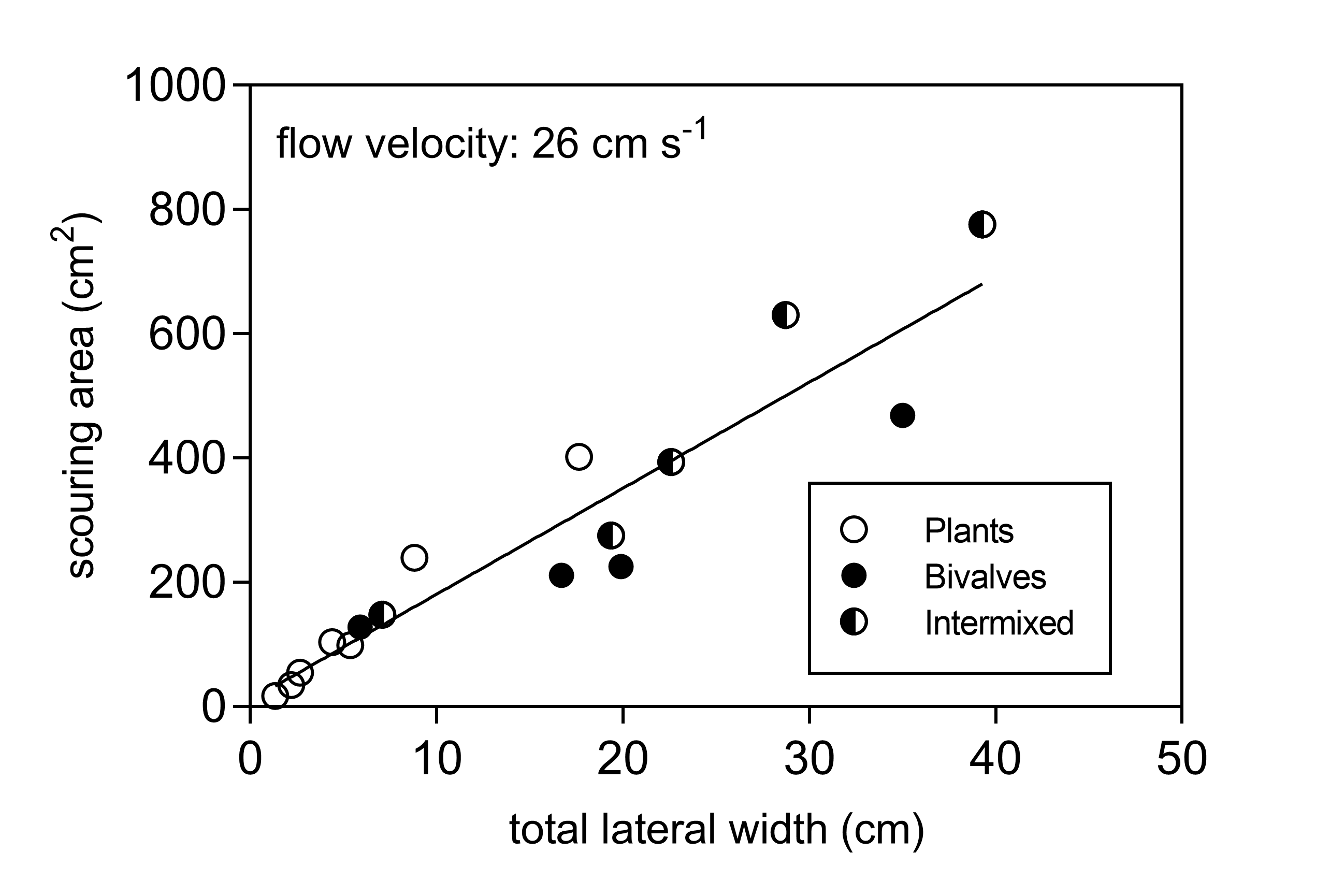

Supplement: S1 Fig — (TIF) [file pone.0222020.s005.tif]

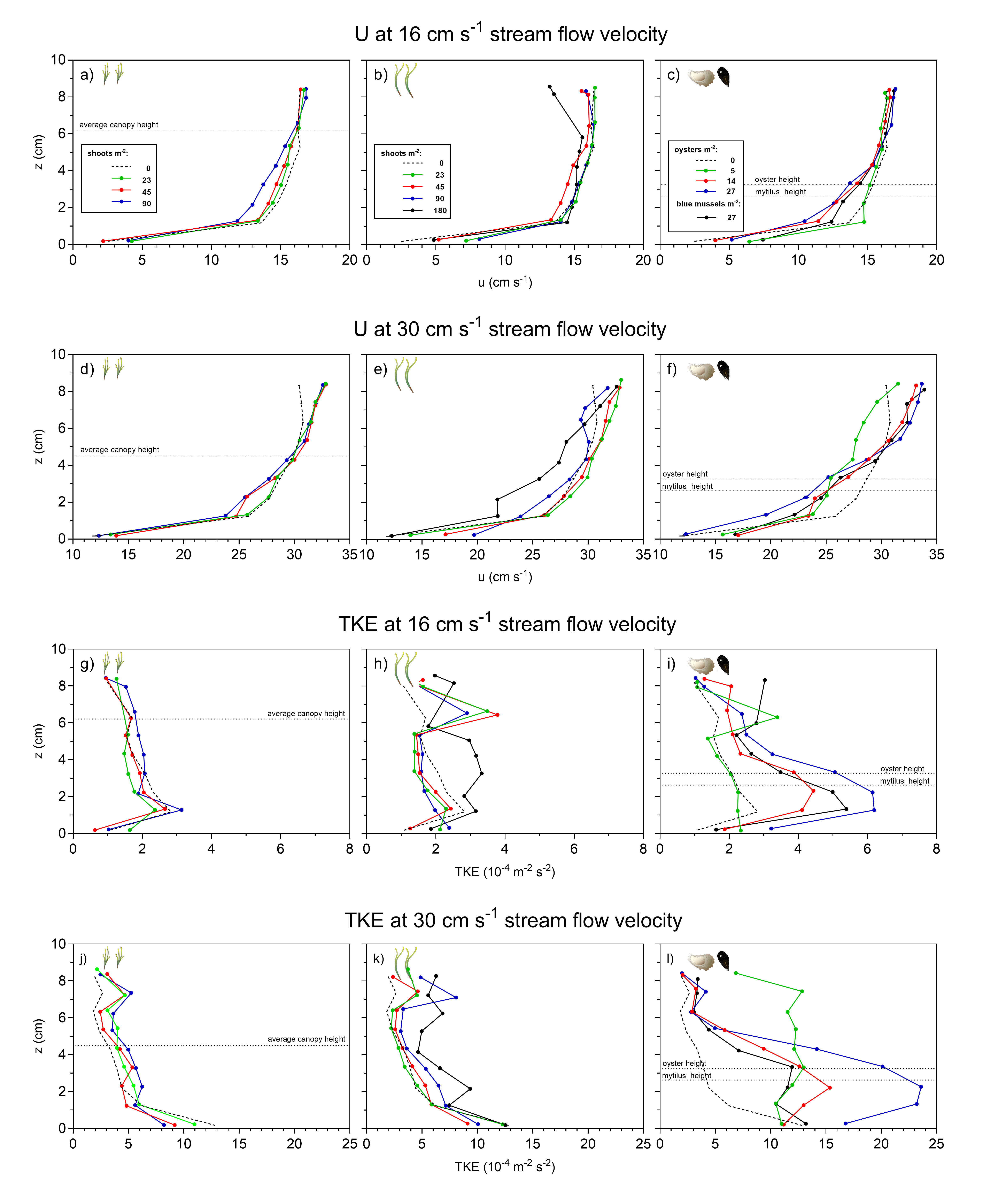

Supplement: S2 Fig — Vertical profiles of flow velocity (U) after test section for different densities of short shoots, large shoots and bivalves at surface flow velocity of 16 cm s-1 (a-c) and 30 cm s-1 (d-f); and vertical profiles of turbulent kinetic energy (TKE) at 16 cm s-1 (g-i) and 30 cm s-1 (j-l). Dashed lines indicate control vertical profiles in bare sand. Symbols courtesy of the Integration and Application Network, University of Maryland Center for Environmental Science (http://ian.umces.edu/symbols/). (TIF) [file pone.0222020.s006.tif]
